# Supplementary material for: Time‑resolved multi-omic analysis of paclitaxel exposure in human iPSC‑derived sensory neurons unveils mechanisms of chemotherapy‑induced peripheral neuropathy
Source: Cell Death Dis. 2026 Feb 10;17(1):211. doi: 10.1038/s41419-026-08445-2 (PMC12921266; doi:10.1038/s41419-026-08445-2)
Supplement: Supplementary file 15 — Dataset 1. [file 41419_2026_8445_MOESM15_ESM.zip › CDD_Open_Data/04_RNA_Seq/Timeline_panel_plots_etc_2023-01-23_1609.zip!/Panel_Plots_etc/Evidence_Plots/72h/evidence_plot_standard contrasts_msigdb_hallmark_72h_PTX_vs_DMSO_ID0_M5932_HALLMARK_INFLAMMATORY_RESPONSE.pdf]

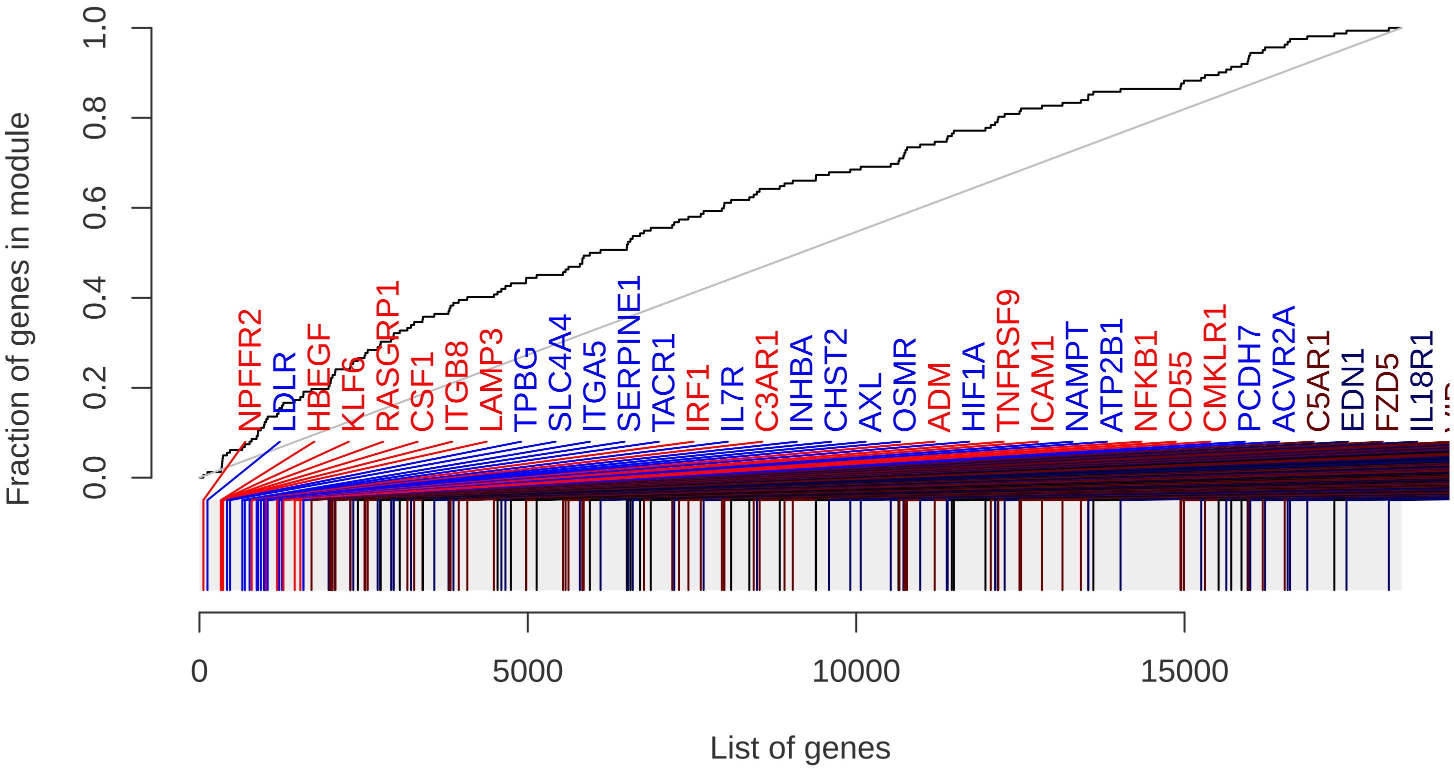
Fraction of genes in module
0.0
0.2
0.4
0.6
0.8
1.0
NPFFR2
LDLR
HBEGF
KLF6
RASGRP1
CSF1
ITGB8
LAMP3
TPBG
SLC4A4
ITGA5
SERPINE1
TACR1
IRF1
IL7R
C3AR1
INHBA
CHST2
AXL
OSMR
ADM
HIF1A
TNFRSF9
ICAM1
NAMPT
ATP2B1
NFKB1
CD55
CMKLR1
PCDH7
ACVR2A
C5AR1
EDN1
FZD5
IL18R1
0
5000
10000
15000
List of genes
